# A geospatial platform to support visualization, analysis, and prediction of tuberculosis notification in space and time

**DOI:** 10.3389/fpubh.2022.973362

**Published:** 2022-09-09

**Authors:** Thang Phuoc Dao, Xuyen Hong Thi Hoang, Doanh Ngoc Nguyen, Nghi Quang Huynh, Thang Tat Pham, Dung Thuy Nguyen, Hoa Binh Nguyen, Nam Hoang Do, Hai Viet Nguyen, Cu Huy Dao, Nhung Viet Nguyen, Hanh My Bui

**Affiliations:** ^1^Hanoi Medical University Hospital, Hanoi, Vietnam; ^2^Hanoi Medical University, Hanoi, Vietnam; ^3^Thuy Loi University, Hanoi, Vietnam; ^4^National Lung Hospital, Hanoi, Vietnam

**Keywords:** tuberculosis, geospatial, visualization, prediction model, artificial intelligence

## Abstract

**Background:**

Tuberculosis has caused significant public health and economic burdens in Vietnam over the years. The Vietnam National Tuberculosis Program is facing considerable challenges in its goal to eliminate tuberculosis by 2030, with the COVID-19 pandemic having negatively impacted routine tuberculosis services at all administrative levels. While the turnaround time of tuberculosis infection may delay disease detection, high transportation frequency could potentially mislead epidemiological studies. This study was conducted to develop an online geospatial platform to support healthcare workers in performing data visualization and promoting the active case surveillance in community as well as predicting the TB incidence in space and time.

**Method:**

This geospatial platform was developed using tuberculosis notification data managed by The Vietnam National Tuberculosis Program. The platform allows case distribution to be visualized by administrative level and time. Users can retrieve epidemiological measurements from the platform, which are calculated and visualized both temporally and spatially. The prediction model was developed to predict the TB incidence in space and time.

**Results:**

An online geospatial platform was developed, which presented the prediction model providing estimates of case detection. There were 400,370 TB cases with bacterial evidence to be included in the study. We estimated that the prevalence of TB in Vietnam was at 414.67 cases per 100.000 population. Ha Noi, Da Nang, and Ho Chi Minh City were predicted as three likely epidemiological hotspots in the near future.

**Conclusion:**

Our findings indicate that increased efforts should be undertaken to control tuberculosis transmission in these hotspots.

## Introduction

Spatial science has developed markedly in recent years due to innovations in surveying and analysis. Geospatial data comprise a highly diverse range of features, including geographical locations, environment features, human data such as postal codes, and satellite-based information, with data coverage ranging from an individual-level to population-level. This wealth of information types creates a data network that scientists can use to analyze and simulate realistic phenomena based on geospatial imagery and information (1, 2). Disease can be caused not only by individual-based risk factors but also by environment-based factors. Exposure to risk factors in terms of environment, living habitats, and mobility trends may significantly contribute to the possibility of a particular disease spreading in a community (3). To address problems of this type, geospatial artificial intelligence (GeoAI) has been developed; this approach has considerable applications for epidemiological studies. In a disease context, GeoAI involves the use of machine learning algorithms, which are supported by geospatial datasets, to explore the impacts of social components, such as population density and migration trends, on community-based disease incidence rates. Such technologies are also beneficial for epidemiologists, as geospatial insights can dramatically strengthen study hypotheses. Maike et al. applied geospatial modeling to identify the association between environmental factors and gestational diabetes mellitus (2). Their study collected postal information, with a sample size comprising nearly 9,000 pregnant women in United States (2). Another study by Lawrence et al. investigated the correlation between environmental pollution and acute asthma events using a dataset including population features, land-use data, distance and topography reports, traffic, and road systems (4). Their study identified a positive correlation between the concentration of chemical gases and the risk of acute asthma events (4).

Tuberculosis (TB) is an airborne disease that can transmit from human to human and causes severe damage to different organs. TB has been a leading health and economic burden worldwide, especially in low–middle-income countries, with a global incidence rate of 127 cases per 100,000 population recorded in 2020 (5). Vietnam remains among the 30 countries with the highest prevalence rates of TB, despite efforts by the Vietnam National TB Program (NTP) to decrease the disease burden over the past decade (5, 6). Given the effects of the COVID-19 pandemic, TB case notification was halted in 2021, which could lead to a potential setback of 8 years on the pathway to the program's goal to end TB by 2030 (7). To promote active case identification, several studies conducted geospatial analyses to determine the distribution of TB cases (8, 9). While TB was frequently detected in low-income areas and those with poor sanitation, the disease prevalence was higher in these areas among subjects who did not have consistent residency status, such as refugees, asylum seekers, and regular immigrants (10). A person who is diagnosed with pulmonary TB and has a high frequency of transportation can cause numerous human-to-human infections and seed new disease clusters (11, 12). The turnaround time of TB infection might also be lengthened, thereby biasing retrospective epidemiology investigations. This issue thus highlights the need for an improved GeoAI application to support healthcare workers in analyzing the spatial characteristics of TB and predicting this disease's epidemiological trends. Thus, the current study was conducted to develop an online GeoAI platform to temporally and geospatially visualize, analyze, and promote TB case notification as well as to predict the TB incidence in community. This GeoAI platform could provide more real-time data relating to demographic information and case distribution, allowing physicians to identify whether a subject is likely to belong to a high-risk group for TB infection.

## Materials and methods

### Study subjects

The participants in our study were bacteriologically confirmed TB patients managed by the Vietnam NTP.

### Study design

This is a cross-sectional study, conducted from the 1st of January 2020 to the 30th of April 2022.

### Study location

The study location comprises all of the facilities that provided TB services at any administrative level and that were managed by the Vietnam NTP.

### Study content

The factors that were deemed to contribute to an increased risk of TB infection are as follows:

- Transmission factor: the number and distribution of TB cases from both temporal and spatial perspectives.
- Individual factor: comorbidities and living habits that increase the risk of TB transmission.
- Mobility factor: internal or external migration of residents. Migration and contact events increase the likelihood of pathogenic human-to-human transmission.
- Climate factor: factors including temperature, humidity, evaporation, radiation, sunshine h, rain, and wind regimen; these depend on the season and, therefore, promote or reduce the likelihood of TB infection.
- Spreading factor: facility-based factors around TB patients that directly affect disease progression, such as high-risk areas (hospitals, industrial clusters, schools, restaurants, tourist areas, crowded areas), population density (typically urban, rural, or mountainous areas), and sensitive areas (boundary areas).
- Socio-economic factor: affects the capacity of TB patients to pay for consultation and treatment. Factors included in the modeling were Gross Domestic Product per person and the rate of poor households in the simulated area.
- Policy factor: administrative solutions by governmental agencies to control the risk of outbreak spread. Intervention policies, such as zoning of epidemic areas and supporting treatment will directly affect the progression of infections.

### Data collection

We collected data from the Vietnam NTP program from the 1st of January 2016 to the 31st of July 2020. Data including the name, age, gender, ethnicity, and physical address of the TB patients were collected for modeling.

### Algorithm features

The cause of TB infection is the transmission of *Mycobacterium tuberculosis* from TB patients to others in the community, which can be reflected by mobility and contact. The transmission magnitude can be considered a function of environmental factors, the infectiousness of TB patients, and the medical status of contacts, including their comorbidities, age, smoking and drinking status, etc. The mobility in the prediction model is indicated by the number of people moving across regions in real time. TB patients would encounter people in an area and their movement to other regions is randomly simulated at all levels of prediction.

Factors relating to population (population density, gender, age), social–economy, climate status, and individual history (i.e., comorbidities and living habits) were included in the predictions to enhance the AI-based simulation, in addition to information from the collected database. Multiple variables were modeled as weighting parameters for users to modify in real time. One such variable is the policy factor, which can indirectly affect the infection magnitude through other factors. To predict TB notification, the GeoAI model includes all the factors described above contributing to TB transmission. The accuracy of the simulation is determined by the quality of the input data; the more detailed the input data is, the more accurate the prediction that can be achieved.

The input parameters which were included during the modelling comprised transmission factor; individual factor; mobility factor; climate factor; socio-economic factor and policy factor. These input parameters were divided into different classes of the Deep-LSTM (Long Short Term Memory) network. The model development was conducted based on the following steps: (1) the above-mentioned parameters were assigned as initial values, a cost function was then developed, (3) the gained values were modified across different steps of looping in order to optimize the developed cost function, and (4) the model were continuously processed until the converged value was achieved. Subsequently, the values which were the inputs of the prediction model was retrieved after the Deep-LSTM model development had been completed. Finally, upscaling methodology was applied to cover all of administrative levels, from district- to nation-level.

We developed an online geospatial platform to support efforts for visualization, analysis, and prediction of TB cases nationwide. The GeoAI platform is managed *via* a uniform resource locator (WebGIS), which requires log-in credentials to access the user interface. The users who registered and provided credentials can log in and review the distribution of TB notifications in space and time. The platform can be viewed in Vietnamese for the convenience of local users in medical practices. Users can access the platform at the following web address: https://geotb.herokuapp.com/.

### Statistical analysis

The distribution of TB cases was visualized while the model's prediction outcomes were both plotted and visualized.

### Ethical approval

All procedures used in this study followed the ethical standards of the Ethical Review Board of the National Lung Hospital (IRB approval No. 48/20/CN-HDDD; approved on 31st December 2020).

## Results

We achieved a sample size of 400,370 TB cases with bacterial evidence. To account for the effects of the COVID-19 pandemic in 2020, we excluded the data from 2020 from the prediction modeling. The data for 2020 were included for epidemiological visualization purposes alone. Based on the study's data, the prevalence of TB in Vietnam was calculated at 414.67 cases per 100,000 population.

**Figure 1:**
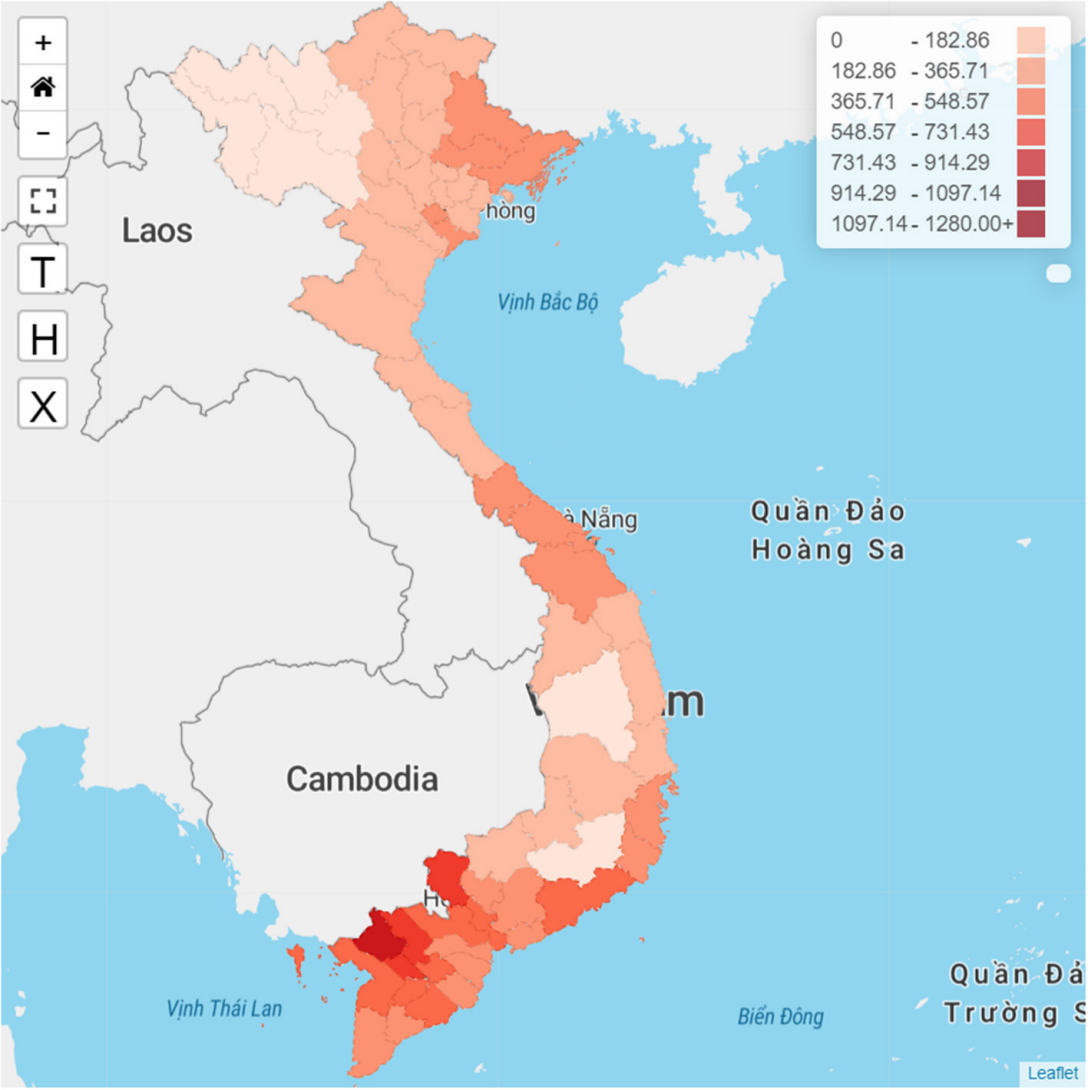
Home screen of the GeoAI platform.

Figure 1 shows the home screen of the GeoAI platform, including interactive elements. The left part of the screen visualizes the distribution of TB notifications using a color spectrum, with darker colors representing more TB cases. Users can enter a full-screen view by clicking on the 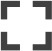 button. To view the map at a province level, users can click on the “T” button while the “H” button and the “X” button represent district and commune levels, respectively. Users can also click on the “+/–” buttons to zoom the map view in and out.

The right part of the screen visualizes TB epidemiological parameters by either population or region, the number and rate of TB cases by time, the distribution of drug-resistant TB cases by age, the distribution of TB cases by treatment outcomes, and the distribution of HIV status in Vietnam. We also designed a filter bar located at the top of the platform that allows users to adjust the data visualization based on either time or administrative levels.

The spatial distribution of TB notification rates in Vietnam at a province level from 2016 to 2020 is illustrated in Figure 1. The TB notifications were relatively higher in the southern part of the country than in the central and northern regions. The TB notifications were notably high in the An Giang, Tay Ninh, Can Tho, Dong Thap, and Soc Trang areas.

**Figure 2:**
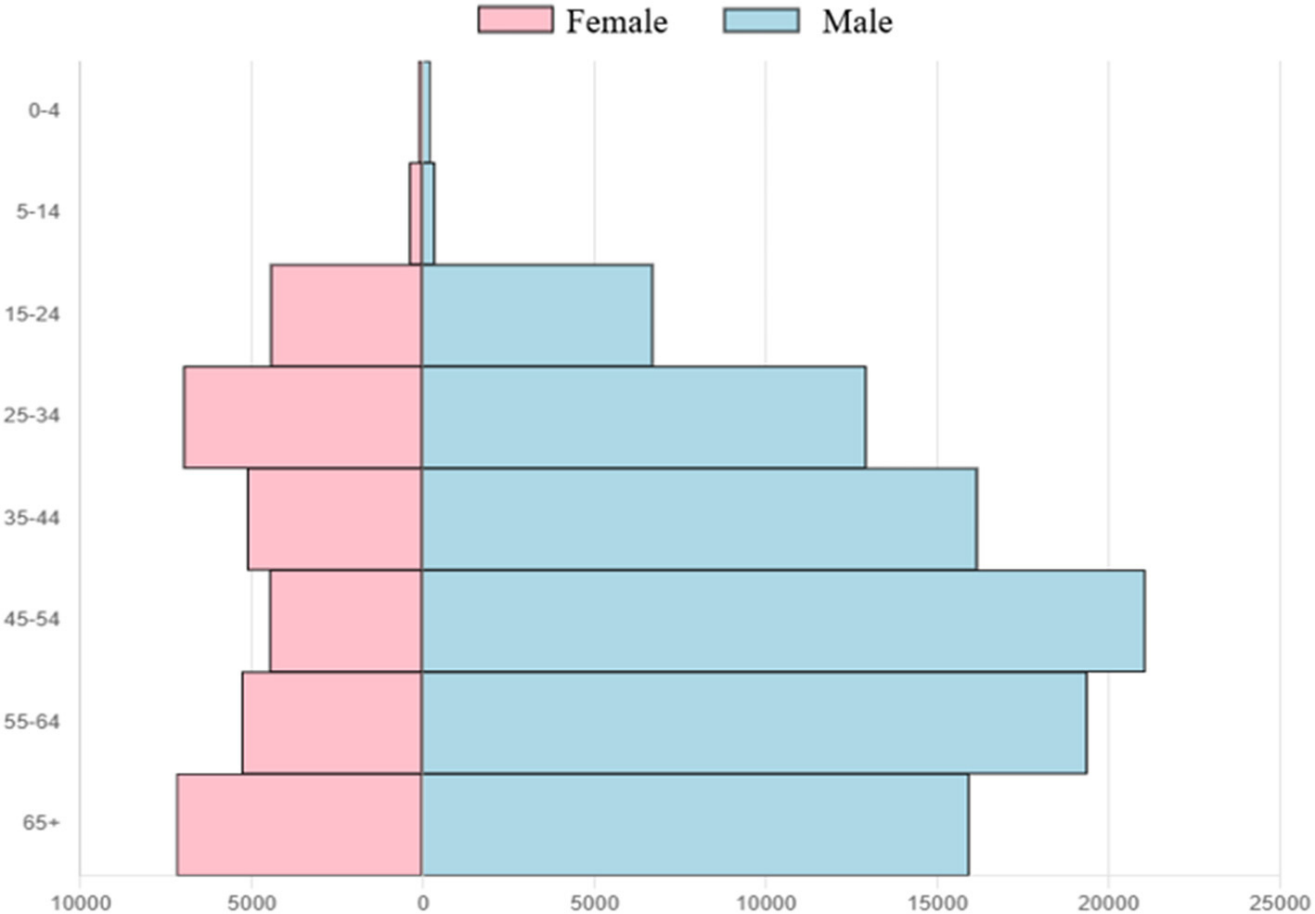
Distribution of TB notifications by sex and age group in Vietnam in 2020.

Figure 2 shows the TB notification rates in Vietnam by sex and age in 2020. TB notifications tended to increase with age, with different TB notification rates recorded between men and women. Adult men had a higher TB notification rate than women of the equivalent age groups, with the lowest male-to-female case ratio of 1.5:1 in the 15–24 age group and the highest ratio of 4.7:1 in the 45–54 age group.

**Figure 3:**
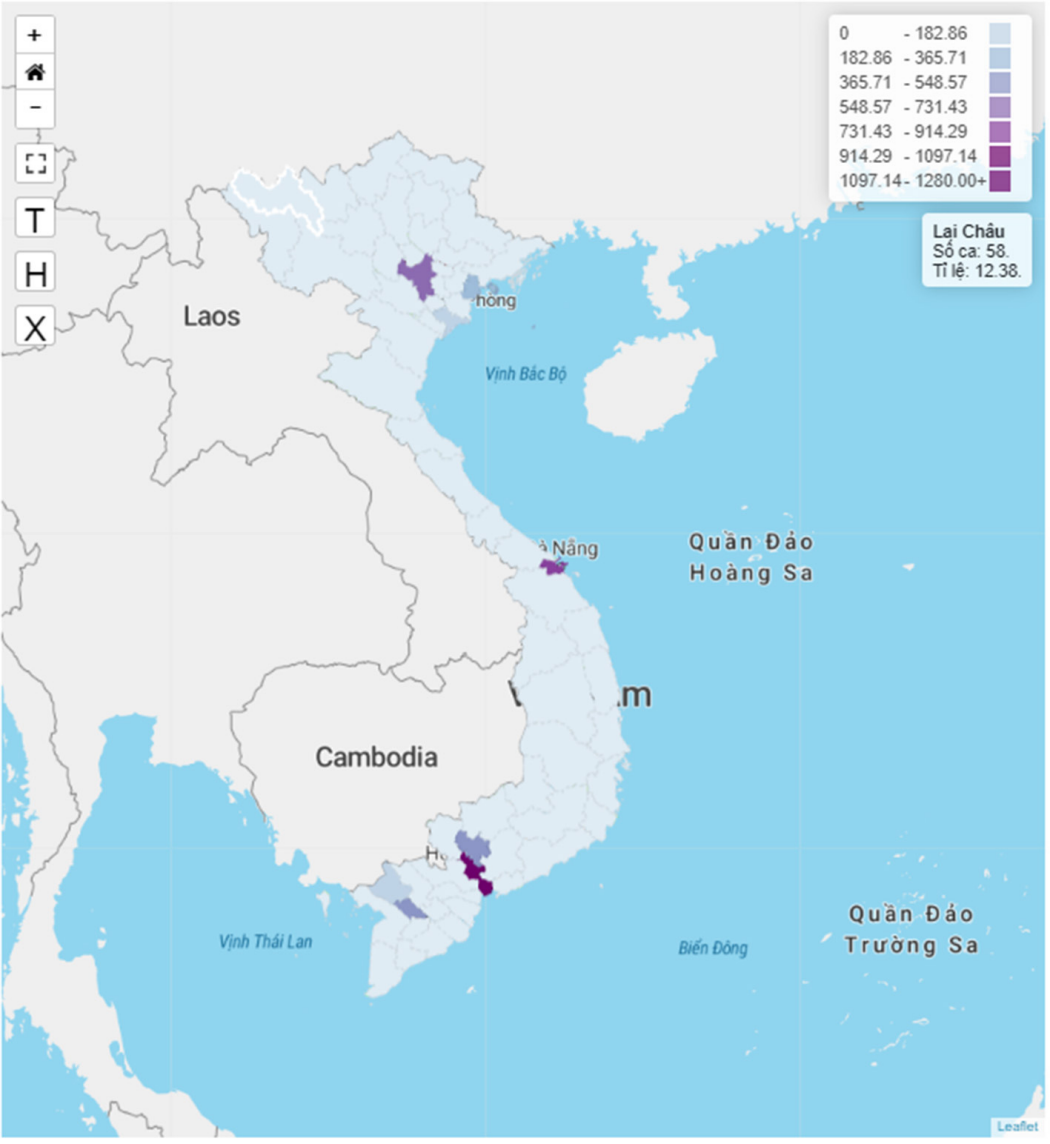
Prediction of TB hotspots in Vietnam.

Figure 3 illustrates the spatial predictions of TB notification in Vietnam based on our model. With all the transmission factors included in the model, three were three cities which were predicted as major TB hotspots, namely Ha Noi, Da Nang, and Ho Chi Minh City.

## Discussion

Our findings indicate that TB notifications in Vietnam show spatial heterogeneity, as demonstrated by the spatial clustering of notifications and predicted hotspots in certain provinces and cities. TB notification rates tend to increase with age and men have higher TB notification rates than women in all age groups.

The high TB rate notification clusters are concentrated in the south of the study area, especially in the southwestern part of the country. This result is consistent with the findings of the second TB prevalence survey in Vietnam, in which TB prevalence was found to be higher in the south compared to the central and northern areas of Vietnam (13). The predicted TB hotspots in the model are Vietnam's three main cities, which are Ha Noi, Da Nang, and Ho Chi Minh City. With the rapid pace of migration and urbanization in Vietnam during the past decade, almost half of the migration in the country was from rural areas to cities (14). Our predictions suggest that TB patients could move to major cities, thus transmitting the infection and creating TB hotspots in these areas. These results are in line with other studies from Zimbabwe (15, 16), where TB hotspots and clusters can be found in urban areas with large populations.

Spatial analysis of TB notification distribution shows that TB is heterogeneous in both time and space, meaning that TB control strategies should be individualized for each area; thus, provinces or regions with high TB rates require increased control efforts compared to those with low TB rates. Areas that are hotspots or clusters of TB cases should be subjects for active TB case identification and innovative interventions. Healthcare policymakers should thus focus on strengthening TB prevention and control measures in these hotspots to mitigate the transmission of this disease.

In addition to the above findings, our study also has certain limitations. Firstly, our main input data source is the TB notification data in Vietnam; due to the effects of the COVID-19 pandemic, the notification data do not accurately represent the TB caseload in Vietnam during the pandemic period, thus greatly affecting the accuracy of our predictions. Secondly, we used aggregated notification data from provincial-level TB hospitals. With the high migration rates in Vietnam, TB patients may have visited more than one provincial TB hospital, thus resulting in duplicate entries. Thirdly, the TB notification system does not include data from TB cases whose treatment was initiated in the private sector. Lastly, the current modeling sample excluded data from 2020 and thus does not fully reflect the most up-to-date epidemiological situation. These problems might cause bias and subsequently affect the prediction of TB hotspots in our model.

## Conclusion

Our GeoAI platform predicted the distribution of TB hotspots, which are located in the major cities of Vietnam. Our findings provide new insights into the spatial patterns of TB, which is essential for targeted regional TB control interventions. This approach is highly important in lower–middle-income countries such as Vietnam, where available resources for TB control are limited and need to be carefully allocated to areas with higher TB caseloads.

## Data availability statement

The raw data supporting the conclusions of this article will be made available by the authors, without undue reservation.

## Author contributions

TD, HB, and XH conceived of the study. TD, HB, XH, and HaN collected and cleaned data, performed the official statistical analyses, interpreted the results, and wrote the manuscript. DoN, NH, TP, DuN, HoN, ND, CD, and NN provided the critical revision of the manuscript for important intellectual content. All authors contributed to the article and approved the submitted version.

## Conflict of interest

The authors declare that the research was conducted in the absence of any commercial or financial relationships that could be construed as a potential conflict of interest.

## Publisher's note

All claims expressed in this article are solely those of the authors and do not necessarily represent those of their affiliated organizations, or those of the publisher, the editors and the reviewers. Any product that may be evaluated in this article, or claim that may be made by its manufacturer, is not guaranteed or endorsed by the publisher.
